# Multidisciplinary Team Meeting Proposal and Final Therapeutic Choice in Early Breast Cancer: Is There an Agreement?

**DOI:** 10.3389/fonc.2022.885992

**Published:** 2022-06-07

**Authors:** Lucia Bortot, Giada Targato, Claudia Noto, Marco Giavarra, Lorenza Palmero, Diego Zara, Elisa Bertoli, Arianna Dri, Claudia Andreetta, Gaetano Pascoletti, Elena Poletto, Stefania Russo, Luca Seriau, Mauro Mansutti, Carla Cedolini, Debora Basile, Gianpiero Fasola, Marta Bonotto, Alessandro Marco Minisini

**Affiliations:** ^1^Department of Medicine (DAME), University of Udine, Udine, Italy; ^2^Department of Medical Oncology, Academic Hospital of Udine, Udine, Italy; ^3^Department of Oncology, Ospedale Santo Spirito, Casale Monferrato, Italy; ^4^Department of Medical Oncology, Aviano Oncology Reference Center, Istituto di Ricovero e Cura a Carattere Scientifico (IRCCS), Aviano, Italy; ^5^Breast Surgery, Department of Medicine (DAME), University Hospital of Udine, Udine, Italy; ^6^Department of Medical Oncology, San Giovanni di Dio Hospital, Crotone, Italy

**Keywords:** multidisciplinary team (MDT), early breast cancer (EBS), breast unit, chemotherapy - oncology, endocrine therapy, elderly patients, clinical decision

## Abstract

**Background:**

A multidisciplinary team meeting (MDM) approach in breast cancer (BC) management is a standard of care. One of the roles of MDMs is to identify the best diagnostic and therapeutic strategies for patients (pts) with new diagnosis of early BC. The purpose of this study was to define whether there was an agreement between the planned program (i.e., MDMs-based decision) and that actually applied. In addition, the study explored factors associated with discordance.

**Methods:**

We conducted a retrospective study of a consecutive series of 291 patients with new diagnosis of early BC, discussed at MDMs at the University Hospital of Udine (Italy), from January 2017 to June 2018. The association between clinico-biological factors and discordance between what was decided during the MDMs and what was consequently applied by the oncologist was explored through uni- and multivariate logistic regression analyses.

**Results:**

The median age was 62 years (range 27–88 years). Among invasive early BC patients, the most frequent phenotype was luminal A (38%), followed by luminal B (33%), HER2-positive (12%), and triple-negative (5%). *In situ* carcinoma (DCIS) represented 12% of cases. The median time from MDM discussion to first oncologic examination was 2 weeks. The rate of discordance between MDM-based decision and final choice, during a face-to-face consultation with the oncologist, was 15.8% (46/291). The most frequent reason for changing the MDM-based program was clinical decision (87%). Follow-up was preferred to the chemotherapy (CT) proposed within the MDMs in 15% of cases, and to the endocrine therapy (ET) in 39% of cases (among these, 44.5% had a diagnosis of DCIS). Therapeutic change from sequential CT-ET to ET alone was chosen in 16/46 pts (35%): among these patients, seven had a luminal B disease and six had an HER2-positive disease. On univariate analysis, factors associated with discordance were values of Ki-67 14%–30% (OR 3.91; 95% CI 1.19–12.9), age >70 years (OR 2.44, 95% CI 1.28–4.63), housewife/retired status (OR 2.35, 95% CI 1.14–4.85), polypharmacy (OR 1.95; 95% CI 1.02–3.72), postmenopausal status (OR 4.15; 95% CI 1.58–10.9), and high Charlson Comorbidity Index (OR 1.31; 95% CI 1.09–1.57). The association with marital status, educational level, alcohol and smoke habits, presence of a caregiver, parity, grading, histotype and phenotype, and stage was not statistically significant. On multivariate analysis, only Ki-67 value maintained its statistical significance.

**Conclusion:**

The results of our study could be useful for enhancing the role of MDMs in the clinical decision-making process in early BC.

## Introduction

Breast cancer (BC) remains the most commonly occurring malignant tumor in women worldwide and represents the second cause of cancer-related death (1).

BC is a highly heterogeneous disease, and over the last decades significant improvements have been achieved in anticipation and accuracy of radiological diagnosis, biomolecular classification precision, and effectiveness of local treatments (2). Therefore, several options for systemic treatment of early breast cancer (EBC) and metastatic breast cancer (MBC) are currently available, and the therapeutic approach should be tailored to the individual patient.

The complexity in the management of BC increased dramatically along with the development of precision medicine. For this reason, diagnostic and decision-making processes require the integrated expertise of different healthcare professionals, such as medical oncologists, surgeons, radiologists, radiation oncologists, pathologists, palliative care specialists, and breast nurses.

Hence, multidisciplinary team meeting (MDM)-based care is in widespread use nowadays in Western countries, and it is recommended in several guidelines (3–5).

Despite the limited number of randomized controlled studies, there is growing evidence that the MDM approach is crucial in making decisions about adjuvant treatments since it provides meaningful improvements in adherence to evidence-based medicine with a better patient clinical outcome, treatment-related satisfaction, and quality of life (6–12).

Furthermore, MDM discussion enhances professional expertise, encourages the enrollment in clinical trials (8, 13), and increases the number of patients receiving neoadjuvant and adjuvant strategies (11, 14), minimizing the time between diagnosis and treatment (15). On the other hand, it seems to reduce medical costs, preventing unnecessary diagnostic procedures (16).

In the great part of MDMs, decisions are commonly based on reported clinical data and patients do not take part to the discussion (17); thus, some patient-center information such as performance status, social vulnerability factors, and preferences might be underestimated. In fact, in the major international guidelines, there is no clear recommendation about what specific clinical records have to be presented during MDM discussions (3, 4).

Accordingly, compliance with MDM recommendations could be influenced by several factors including, among others, age, comorbidity, and polypharmacy, as highlighted by Ring et al., which reported a higher rate of discordance among older BC patients, especially in adjuvant settings (18).

There is lack of studies evaluating the consistency between final decision on adjuvant strategies in early BC and MDM recommendations. The aim of this study was to investigate the rate of discrepancy between recommendations issued by MDMs and final face-to-face decision about medical adjuvant strategies for EBC.

The aim of this study was to investigate the rate, the reasons and factors associated with discordance between MDM decision and what currently applied by the oncologist after the face-to face examination with patient.

## Materials and Methods

### Study Design

This observational, retrospective, no-profit, single-center cohort study analyzed data of 291 consecutive EBC patients discussed at MDMs at the Academic Hospital of Udine (Italy), from January 2017 to June 2018. The study was conducted under the Declaration of Helsinki, and informed consent was obtained for all enrolled patients for the use of clinical data, rendered anonymous, for purposes of clinical research, epidemiology, training, and study of diseases. The study was approved by the Ethical Review Committee.

### Data Source

Clinicopathological information was collected from electronic health records according to strict privacy standards.

We defined tumors as estrogen receptor (ER) or progesterone receptor (PgR) positive when they had an expression of ≥1%, HER2-positive when the immunohistochemistry survey (IHC) had a 3+ score or fluorescent *in situ* hybridization (FISH) had a HER2/CEP17 ratio ≥2.

BC subgroups were defined as follows: luminal A (ER- or PR-positive, HER2-negative, Ki-67 <14%), luminal B (ER- or PR-positive, HER2-negative, Ki-67 ≥14%), luminal HER2 (HER2-positive and ER- or PR-positive), HER2-positive (ER- and PR-negative, HER2-positive), and triple-negative (ER- and PR-negative, HER2-negative) (19).

Pathological stage was defined according to the eighth edition of the TNM staging system.

Comorbidities were evaluated through the Charlson Comorbidity Score Index (CCS), which had previously been validated as a predictor of survival (20, 21).

All confirmed or suspected diagnoses of early BC were discussed at the MDM, which is regularly scheduled once a week and involves breast surgeons, medical oncologists, radiologists, radiation oncologists, nuclear medicine specialists, histopathologists, plastic surgeons, breast care nurses, and data managers.

A template of patients’ MDM record is shown in **Supplementary Material**.

At our Institution, EBC cases are presented at the MDM both at diagnosis to define the best therapeutic approach (immediate surgery versus neoadjuvant treatment) and after radical surgery to discuss and plan any adjuvant strategies. Each tumor board indication is subsequently registered and available electronically on patients’ personal records. A team of medical oncologists with expertise in breast cancer attended the MDM; it should be taken into consideration that, due to organizational reasons, the medical oncologist examining the patient may not have attended the relevant MDM.

MDM recommendations are based on well-recognized national and international guidelines about adjuvant treatment of EBC.

### Statistical Analysis

The study aimed to explore the consistency between the planned medical adjuvant treatment program based on MDM decision and the final clinical choice made by medical oncologists after discussion with the patient. The primary endpoint was the rate of discrepancy between MDM and the final clinical decision. Furthermore, as secondary analysis we evaluated pathological and clinical factors associated with discordance.

Baseline patients’ clinicopathological characteristics, rate, and reasons of discordance were summarized through descriptive analysis. Categorical variables were described through frequency distribution, whereas continuous variables were reported through median range.

The impact of clinicopathological features determining discordance was analyzed with logistic regression.

Predictive factors in terms of disagreement were tested in both univariate and multivariate models by Cox regression with 95% confidence interval (95% CI). A two-sided p ≤ 0.05 was considered statistically significant. The univariate and multivariate models included the following covariates: age, education level, marital status, employment, menopausal status, parity, smoke and alcohol habits, drugs, presence of a caregiver, social impairments, stage, histotype, grading, molecular profile, ECOG PS, and CCS index.

Statistical analysis was performed with STATA (StataCorp, www.stata.com (2015) Stata Statistical Software: Release 14.2. College Station, TX, USA: StataCorp LP).

## Results

### Patients’ Characteristics

A consecutive series of 291 women with early BC discussed at least once during MDMs were included in the analysis. The median age was 62 years (range 27–88), and the great part of the patients (69.4%) were younger than 70 years. Postmenopausal women accounted for 70% of cases.

At BC diagnosis, nearly half of patients were retired or housewives (51.8%), while the other half were currently working (48.20%). Fifty-two percent of enrolled women attended university and graduated; on the contrary, 48% had a lower level of school education. Overall, 72% of patients were married at the time of the diagnosis and 85% of them had one or more children. A caregiver attended the 79% of patients, while only 21% faced the diagnosis of BC alone; social impairments were detected in 12% of cases. Approximately 40% of women were active or former smokers; contrariwise, no smoking history was detected in the other 60%. Concerning alcohol, 37% of patients reported a moderate consumption and 63% a low or zero consumption, while no women declared alcohol abuse.

At diagnosis, nearly 70% of subjects were taking two or less drugs, while 30% were taking three or more concomitant medications. Overall, in the whole cohort, the mean CCS index score was 4.04 (range 2–11).

*In situ* carcinoma (DCIS) was detected in 12% of cases, while, consistently with literature, invasive ductal carcinoma was the most common histology (70.83% of cases). Among invasive carcinomas, 54% of patients had a tumor smaller than 2 cm (T1), 26% between 2 and 5 cm (T2), and nearly 5% larger than 5 cm at diagnosis (T3 and T4). Ki-67 was between 14 and 30 in 8% of cases (14 patients). HR-positive tumors were the most represented subtype (38% were luminal A, 33% luminal B), followed by HR-negative/HER2-positive (12%) and TNBC (5%) (see Section 2.2 for classification details). Lymph node involvement N2 or N3 was detected in 9% of cases, while N0 or N1 disease was diagnosed in most of the patients, respectively 70% and 30%.

A moderately differentiated carcinoma (G2) was found in approximately half of the cases, while a poorly differentiated cancer (G3) and a well-differentiated cancer (G1) were detected in 32% and 18% of patients, respectively.

Additional baseline clinical and pathological characteristics of patients are listed in **Table 1**.

**Table 1:** Baseline patients’ clinicopathological characteristics.

Characteristics		Number of patients	% of patients
**Age**	<70 years	202	69.4
	≥70 years	89	30.6
**Menopausal status**	Premenopausal	87	30.0
	Postmenopausal	203	70.0
**Marital status**	Married	203	71.7
	Unmarried	80	28.3
**Education**	Low degree	133	47.8
	High degree	145	52.2
**Job**	Worker	134	48.2
	Housewife/retired	144	51.8
**Number of children**	0	43	15.0
	≥1	244	85.0
**Smoking**	YES/former smoker	115	40.9
	NO	166	59.1
**Alcool habit**	Low	175	62.7
	Moderate	104	37.3
**Nr drugs**	≤2	201	69.3
	≥3	89	30.4
**Presence of caregiver**	YES	228	79.4
	NO	59	20.6
**Social impairments**	YES	12	4.3
	NO	268	95.7
**Histotype**	Ductal	208	70.8
	Others	84	29.1
**Grade**	G1	52	18.1
	G2	144	50.2
	G3	91	31.8
**Ki-67**	<14 or >30	161	92.0
	14–30	14	8.0
**Biological Profile**	Luminal A	110	37.8
	Lumina B	96	33.0
	HER 2 positive	35	12.0
	Triple negative	16	5.5
**T**	pTis	34	11.7
	pT1	156	54.0
	pT2	75	26.0
	pT3	10	3.5
	pT4	6	2.1
**N**	0–1	200	72.7
	2–3	75	27.7

### Rate and Characteristics of Discordance

The median time from MDM discussion to first oncological examination was 2 weeks, and the rate of discordance between MDM-based decision and final therapeutic choice taken during face-to-face consultation with the oncologist was 15.8% (46/291).

The most frequent reason for changing the MDM-based program was clinical decision (87%), followed by patient’s preference (13%).

Through univariate analyses, age, occupation, menopausal status, polypharmacological therapy, Ki-67, and CCS score emerged as factors associated with discordance.

More specifically, patients >70 years old (OR 2.44, 95% CI 1.28–4.63 p = 0.007), postmenopausal (OR 4.15; 95% CI 1.58–10.9 p = 0–004), and housewife/retired status (OR 2.35, 95% CI 1.14–4.85 p = 0.021) experienced a higher rate of discordance.

Moreover, three or more concomitant medications and higher CCS index were associated with change of MDM-treatment plan performed by the oncologist, with OR 1.95; 95% CI 1.02–3.72 p = 0.043 and OR 1.31; 95% CI 1.09–1.57 p = 0.004, respectively.

Among disease-associated factors, only a Ki-67 value between 14% and 30% was associated with discordance (OR 3.91; 95% CI 1.19–12.9 p = 0.024) and maintained its statistical significance on multivariate analysis (OR 5.04; 95% CI 1.23–20.56 p = 0.024).

Association with marital status, educational level, alcohol and smoke habits, presence of a caregiver, parity, grading, histotype and phenotype, and stage resulted to be not statistically significant.

Predictive factors of discordance according to univariate and multivariate Cox models are listed in **Table 2**.

**Table 2:** Factors associated with discordance through univariate and multivariate analyses.

Characteristics		Number of patients	Univariate analysis (HR, 95% CI)	p	Multivariate analysis (HR, 95% CI)	p
**Age**	≥70 years	89	2.44 (1.28–4.63)	**0.007**	2.99 (0.64–13.95)	0.163
**Menopausal status**	Postmenopausal	203	4.15 (1.58–10.90)	**0.004**	3.92 (0.58–18.74)	0.179
**Marital status**	Unmarried	80	1.02 (0.49–2.10)	0.962		
**Education**	High degree	145	0.63 (0.31–1.26)	0.184		
**Job**	Housewife/retired	144	2.35 (1.36–4.85)	**0.021**	2.48 (0.68–9.12)	0.171
**Number of children**	≥1	244	1.73 (0.46–2.97)	0.736		
**Smoking**	NO	166	0.87 (0.44–1.70)	0.686		
**Alcohol habit**	Low	175	0.76 (0.39–1.48)	0.418		
**Number of drugs**	≥3	89	1.95 (1.02–3.72)	**0.043**	0.30 (0.07–1.28)	0.104
**Presence of caregiver**	YES	228	0.86 (0.40–1.86)	0.699		
**Social impairments**	YES	12	1.14 (0.24–5.40)	0.869		
**Histotype**	Others	84	1.59 (0.82–3.10)	0.169		
**Grade**	G1/G3	143	1.47 (0.78–2.80)	0.237		
**Ki-67**	<14 or >30	161	0.26 (0.08–0.83)	**0.024**	0.20 (0.05–0.81)	**0.024**
	14–30	14	3.91 (1.19–12.87)	**0.024**		
**Biological profile**	Others		1.15 (0.56–2.37)	0.703		
**T**	Others		1.04 (0.52–2.07)	0.909		
**N**	0–1	252	0.55 (0.28–1.10)	0.090	0.28 (0.98–0.80)	0.018
**CCS score**	High		1.31 (1.09–1.57)	**0.004**	0.88 (0.09–8.80)	0.913

Bold values are p ≤ 0.05, considered statistically significant.

Follow-up was preferred to chemotherapy (CT) proposed within the MDMs in 15% of cases and to endocrine therapy (ET) in 39% of cases: intriguingly, among these, 44.5% had a diagnosis of DCIS.

Therapeutic change from sequential CT-ET to ET alone was chosen in 16/46 patients (35%): most of them (seven) had a luminal B disease, while six women had a HER2-positive disease.

Focusing on 13% of cases of discordance due to patient's preference, in almost half of these CT was declined in favour of ET, while in 30% of case follow up was preferred to ET; only in 20% of cases patients refused CT and chose follow up.

## Discussion

The increasing complexity in management of cancer care requires a multimodal approach; consequently, meetings involving different specialists progressively gained relevance. Despite the lack of solid evidence, breast MDMs are widely accepted by the medical community; they increase the level of satisfaction of participating, supporting improvement of decision making and overall quality of treatment. They also promote more coordinated patient care and evidence-based treatment decisions (17, 22).

The association between MDM-based treatment plans and improvements in pathological and radiological assessment and an increased number of neoadjuvant and adjuvant strategies proposed have already been described (15, 23). It has also been suggested that tumor boards might increase survival outcomes in different tumors, including BC, although evidence is not conclusive (15, 24). Several studies highlighted the association between multidisciplinary discussion and subsequent changes in treatment plans (15, 25). However, to our best knowledge there is a paucity of evidence describing the concordance between MDM recommendation and what was proposed to the patient.

In our retrospective study, a discordance rate of 15.8% was found between what collegiality proposed during MDMs and what was subsequently applied by the oncologists during a face-to-face consultation with the patient. This discrepancy was predominantly caused by a medical decision based on patients’ clinical and psychological conditions (87% of cases) rather than a patient choice. Intriguingly, a de-escalation of treatment intensity was performed in most cases, with adjuvant ET preferred to sequential CT and ET proposed within the MDM in 35% of women. Furthermore, clinical follow-up based on scheduled physical examination and breast imaging was chosen instead of CT and ET in 15% and 39% of patients, respectively.

Focusing on cases in which follow-up was preferred to ET, 44.5% were DCIS, for which the clinical impact of adjuvant tamoxifen should be discussed with each patient for a correct risk and benefit balance, particularly in elderly women.

As emerged by univariate analysis, high CCS index, concomitant medications, age older than 70 years, and consequent postmenopausal status were factors associated with discordance. Intuitively, patients with several comorbidities and advanced age are considered less likely to be offered active anticancer treatment, especially in adjuvant settings. Hence, post-MDM treatment de-escalation observed in our series could be related to new information about comorbidities highlighted during the oncological examination, in line with other studies (26, 27). Blazeby et al., in a series of 271 upper gastrointestinal cancer patients, observed that 15.1% of MDM decisions were not implemented and patients mostly received a more conservative treatment due to new data concerning health status (26). Similarly, in another study evaluating 201 MDM decisions in colorectal patients, the discordance rate was 10.0% and a more conservative treatment was chosen in all cases mainly due to patients’ related factor (comorbidities or personal decisions) (27).

In addition, in the great part of centers, patients do not participate in MDMs and thus the panel decision is taken on available clinical record data and without considering patients’ preferences.

On the other hand, it might be more difficult for MDM members to define a clear treatment plan in multi-pathological patients and, when an agreement is reached, recommendations are less likely concordant with anticancer treatment guidelines for patients with comorbidities and older age (28). As confirmed in a series of breast cancer patients older than 70 years, age might be an independent factor associated with a lower rate of adjuvant CT proposed within the tumor board, especially older than 80 years (29). A correlation between older age and reduced adjuvant CT performed was also highlighted in a French study (30).

Patients’ social status, psychological conditions, and caregiver attendance are additional elements of complexity that might not always be adequately discussed during MDMs, emerging subsequently during oncological visit and supporting the decision of a lower intensity of care. Indeed, in a survey performed by the Korea Breast Cancer Society, only 2.1% of meetings also considered patients’ psychosocial issues (31).

When considering disease-associated characteristics, in our series Ki-67 between 14% and 30% emerged as the only factor associated with discordance, in both uni- and multivariate logistic regressions. Tumor proliferation activity is a well-known prognostic factor, and in hormone-positive tumors the cutoff of 20% (previously 14%) separates luminal A and B cancers (32). At the state of the art, it is not possible to define a specific threshold value above which BC is considered highly proliferative, to predict the efficacy of adjuvant CT or ET. Thus, in the “gray zone” between 14% and 30%, other clinicopathological factors might impact the medical decision on adjuvant treatment, increasing the approach variability and justifying the post-MDM discordance observed in our series.

The present study has several limitations, the first of which are the retrospective design and the small number of patients enrolled. Furthermore, despite that in our center both pre- and postoperative breast cancer cases are routinely discussed at MDMs, in this report we focused only on patients who previously underwent radical surgery. Although the tumor board indication is registered and available electronically, the physician taking charge of the patient might have not attended the multidisciplinary discussion and might disagree with the board’s decision.

This interindividual variability could be a further element explaining the discordance between what was decided during the MDMs and what was consequently applied by the oncologist. Indeed, the panel’s opinion should be interpreted as a recommendation and not as the final decision, which is up to the oncologist who takes charge of the patients.

Genomic testing was not available in clinical practice at the time of the data collection of the present study. Several studies highlighted its utility in predicting benefit from chemotherapy and established the importance of integrating these tools in EBC management, hence in MDM discussions.

Notwithstanding the limitation cited above, our study provides a pragmatic real-life view on breast cancer decision-making processes.

## Conclusions

MDM is gaining momentum as a gold-standard resource in BC healthcare management since it improves decision-making processes, empowers medical team professional skills, and ensures comprehensive taking charge of patients. In our opinion, every effort should be made to promote MDM development, improving their reliability and cost-effectiveness. However, it should be recognized that discrepancy could be forecast between MDM recommendation and final clinical decision with the patient. This study suggests that, in order to optimize time and resources, vulnerability factors such as age, comorbidity, and social impairments should be properly assessed, routinely presented, and discussed during MDMs.

Furthermore, the participation of a greater number of professionals; the integration of psychologists, onco-geriatricians, and social care professionals in the MDM’s team; and the systematic adoption of geriatric assessment scale could be meaningful to improve clinical outcomes.

## Data Availability Statement

The raw data supporting the conclusions of this article will be made available by the authors, without undue reservation.

## Ethics Statement

The studies involving human participants were reviewed and approved by Comitato Etico Unico Regionale - CEUR FVG. Written informed consent for participation was not required for this study in accordance with the national legislation and the institutional requirements.

## Author Contributions

LB, MG, MB, and AM designed the study. LB, GT, and CN wrote the initial manuscript. GT, MG, LP, DZ, and EB performed the data collection and cleaning. DB performed the data analysis. All authors contributed to the article and approved the submitted version.

## Funding

Fees for open access were provided by Azienda Sanitaria Universitaria Friuli Centrale (ASUFC).

## Conflict of Interest

The authors declare that the research was conducted in the absence of any commercial or financial relationships that could be construed as a potential conflict of interest.

## Publisher’s Note

All claims expressed in this article are solely those of the authors and do not necessarily represent those of their affiliated organizations, or those of the publisher, the editors and the reviewers. Any product that may be evaluated in this article, or claim that may be made by its manufacturer, is not guaranteed or endorsed by the publisher.
