# Supplementary material for: Multidisciplinary Team Meeting Proposal and Final Therapeutic Choice in Early Breast Cancer: Is There an Agreement?
Source: Front Oncol. 2022 Jun 7;12:885992. doi: 10.3389/fonc.2022.885992 (PMC9209643; doi:10.3389/fonc.2022.885992)
Supplement: Supplementary file 1 [file Table_1.docx]

**Multidisciplinary evaluation**

| **Clinico-pathological informations** | |
| --- | --- |
| Menopausal status |  |
| Comorbidities |  |
| Concomitant drugs |  |
| Cancer familiy history |  |
| Diagnostic imaging |  |
| Histotype |  |
| ER |  |
| PgR |  |
| Ki67 |  |
| HER2 (IHC score) |  |
| HER2 (FISH) |  |
| Stage |  |
| Diagnostic program |  |
| Neoadjuvant treatment |  |
| Type of surgery |  |
| Histotype |  |
| pTNM |  |
| DCIS |  |
| LVI |  |
| ER |  |
| PgR |  |
| Ki67 |  |
| HER2 (IHC score) |  |
| HER2 (FISH) |  |
| Surgical margins |  |
| **Postoperative program** | |
|  | |

ER:estrogen receptor; PgR:progesterone receptor; DCIS:ductal carcinoma in situ; LVI: Lymphovascular invasion
